# Warm Autoimmune Hemolytic Anemia Unmasked by Blood Transfusion in a Patient With Prostate Cancer: A Case Report

**DOI:** 10.7759/cureus.95033

**Published:** 2025-10-21

**Authors:** Jesheen Mann, Sihab Uddin, Rutbah Khairati, Mubshra Tariq, Zill-e- Huma

**Affiliations:** 1 Internal Medicine, George Eliot Hospital NHS Trust, Nuneaton, GBR; 2 Internal Medicine, Northampton General Hospital NHS Trust, Northampton, GBR

**Keywords:** blood transfusion, direct antiglobulin test, prostate cancer, secondary autoimmune hemolysis, warm autoimmune hemolytic anemia

## Abstract

We report the case of a 65-year-old man with non-metastatic prostate cancer who presented with symptomatic anemia (Hb 66 g/L). With no bleeding source and normal iron studies, anemia of chronic disease was initially suspected. Following transfusion of one unit of red blood cells, he developed clinical and biochemical features of hemolysis. The direct antiglobulin test (DAT) was strongly positive (IgG +4, C3 +3), confirming warm autoimmune hemolytic anemia (AIHA). Further transfusions were administered under steroid cover. Autoimmune serology revealed positive antinuclear antibodies (ANA) and markedly elevated dsDNA by enzyme-linked immunoassay (ELISA) but negative *Crithidia* assay, indicating low-affinity antibodies and no clinical features suggestive of systemic lupus erythematosus (SLE). No monoclonal proteins were detected, and immunoglobulin profile showed polyclonal hypergammaglobulinemia. The patient had no features to suggest lymphoproliferative disorders. He responded to high-dose prednisolone and supportive treatment without further transfusions. While warm AIHA is more frequently reported in association with chronic lymphocytic leukemia (CLL) or SLE, its occurrence in patients with solid organ malignancies such as prostate cancer is rare. Given the absence of systemic autoimmune disease and drug exposure, and in the context of known non-metastatic prostate cancer, this case of warm AIHA is best explained by malignancy-associated immune dysregulation.

## Introduction

Autoimmune hemolytic anemia (AIHA) is an infrequent condition, reported in 0.8-3 cases per 100,000 population per year, characterized by immune-mediated destruction of erythrocytes [1]. Although a lot has been learned about how the disease causes hemolysis, the exact reasons why it develops remain unclear and are likely due to multiple factors [1]. The diagnosis of AIHA is confirmed with a positive direct antiglobulin test (DAT), IgG, and C3 testing [2]. The risk factors associated with this condition are malignancy, autoimmune diseases, or drugs [1,3]. The incidence of AIHA increases with age, but diagnosis is often delayed in elderly patients due to the presence of coexisting anemia-related conditions. While lymphoproliferative disorders and systemic lupus erythematosus (SLE) are known triggers, the development of AIHA in the context of solid tumors, particularly prostate cancer, is uncommon [4-6]. Emerging evidence suggests that in patients with solid organ malignancies, immune dysregulation induced by the tumor microenvironment may precipitate autoantibody formation. This case report presents a rare instance of warm AIHA in a patient with non-metastatic prostate cancer, unmasked following red blood cell transfusion. It emphasizes the diagnostic complexity and therapeutic considerations associated with autoimmune hemolysis in the context of prostate cancer, which is rarely reported in the literature, and highlights the need for heightened clinical suspicion and judicious use of immunohematological testing.

## Case presentation

A 65-year-old male with a medical background including non-metastatic prostate cancer, ischemic heart disease, and Alzheimer’s disease presented with symptoms such as fatigue and noticeable pallor, but without any overt bleeding. His initial hemoglobin was alarmingly low, which led to an investigation for potential causes of anemia. Upper gastrointestinal endoscopy excluded a bleeding source, prompting further evaluation of iron metabolism to explain the etiology of the anemia. Serum iron and total iron-binding capacity (TIBC) were within normal limits, consistent with anemia of chronic inflammation. Transferrin saturation was elevated, reflecting efficient iron utilization. These results effectively ruled out iron-deficiency anemia and instead suggested an alternative etiology. 

**Figure 1:**
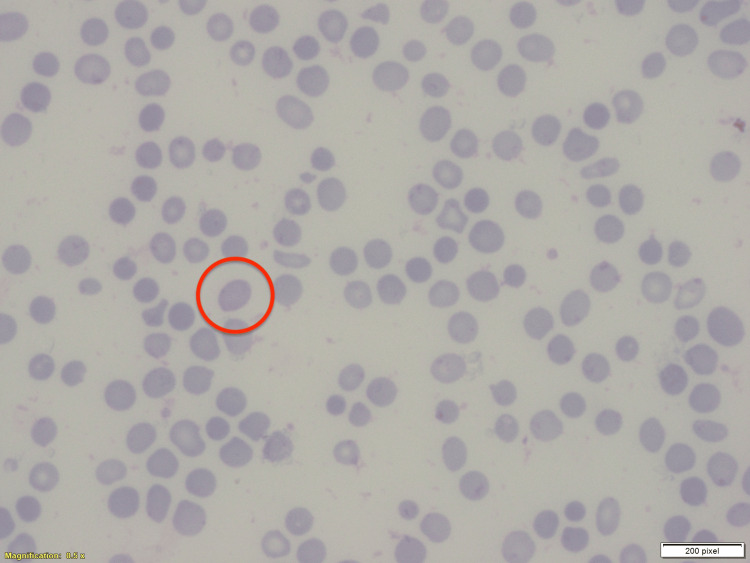
Peripheral blood smear showing polychromasia outlined with a red circle.

**Figure 2:**
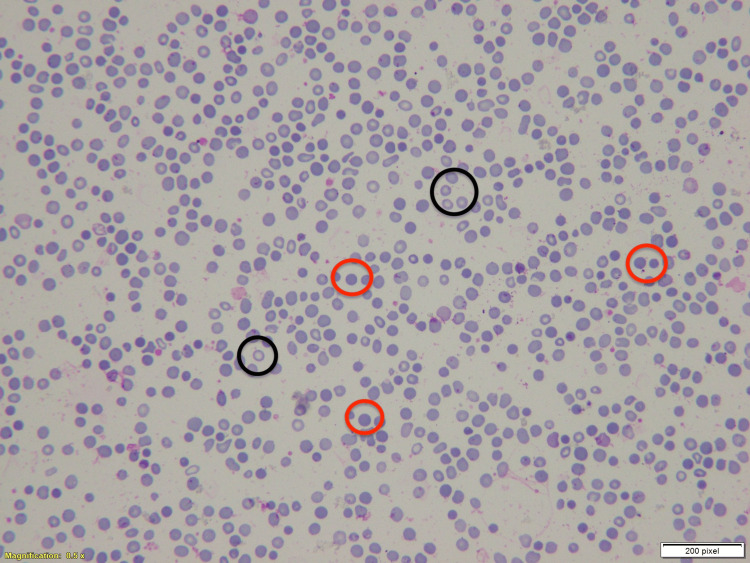
Peripheral blood smear showing spherocytes. Red circles highlight spherocytes, which are smaller, round, and lack central pallor, in contrast to the normal biconcave erythrocytes marked with black circles. These findings are characteristic of warm autoimmune hemolysis.

This led to an initial provisional diagnosis of anemia of chronic disease, a common finding in patients with long-standing malignancy or chronic inflammatory states. However, the clinical picture quickly evolved following the transfusion of one unit of red blood cells. A few days after transfusion, the blood tests were repeated to monitor the hemoglobin trend, which showed biochemical evidence strongly suggestive of hemolysis with elevated lactate dehydrogenase (LDH), undetectable haptoglobin, and raised total and unconjugated bilirubin (Table 1). Polychromasia with spherocytes was visualized on the blood film (Figures 1, 2). 

**Table 1:** Laboratory evaluation performed after red blood cell transfusion, confirming warm autoimmune hemolysis. LDH: lactate dehydrogenase; DAT: direct antiglobulin test; ELISA: enzyme-linked immunoassay; ENA: extractable nuclear antigen; TIBC: total iron-binding capacity

Test	Results	Reference range
Hemoglobin	66 g/L	130-180g/L
White Cell Count	9.0 x 10^9^/L	4.0–11.0 × 10⁹/L
Neutrophils	5.94 x 10^9^/L	2.0–7.5 × 10⁹/L
Lymphocytes	2.12 x 10^9^/L	1.5–4.0 × 10⁹/L
Platelets	403 x 10^9^/L	150–400 × 10⁹/L
Reticulocyte Count	150 x 10^9^/L	25–100 × 10⁹/L
LDH	453 U/L	125 – 220 U/L
Haptoglobin	<0.1 g/L	0.3 – 2.0 g/L
Total Bilirubin	47 μmol/L	<21 µmol/L
Conjugated Bilirubin	19 μmol/L	<5 μmol/L
DAT (IgG)	Strongly positive +4	Negative
DAT (C3)	Positive +3	Negative
Anti ds-DNA (ELISA)	>666.9 IU/mL	<100 IU/mL
Rheumatoid Factor	<10 IU/mL	<20 IU/mL
ENA Panel	Negative	Negative
IgG	16.6 g/L	7.0 – 16.0 g/L
IgA	4.9 g/L	0.7 – 4.0 g/L
IgM	0.26 g/L	0.4 – 2.3 g/L
Serum Protein Electrophoresis	No monoclonal protein detected	
Iron	25 μmol/L	11 - 30 μmol/L
TIBC	46 μmol/L	45 – 72 μmol/L
Transferrin Saturation	54%	20–45%

The reticulocyte count was elevated, indicating an appropriate bone marrow response to hemolysis (Table 1). These changes pointed towards an autoimmune hemolytic process, which was confirmed by a strongly positive DAT. The absence of previous transfusions and lack of new alloantibodies on antibody screening ruled out both acute and delayed hemolytic transfusion reactions. Anti-IgG and C3 were positive, consistent with warm AIHA (Table 1). 

The complete blood count demonstrated isolated anemia without lymphocytosis, thrombocytosis, or thrombocytopenia, suggesting no overt hematological malignancy. As a part of the workup for warm AIHA, autoimmune serology was performed, which revealed that antinuclear antibodies (ANA) were positive with a mixed homogenous and speckled pattern (Table 1). Although the anti-double-stranded DNA (dsDNA) was strongly positive by ELISA, the more specific *Crithidia luciliae* assay was negative, suggesting the presence of low-affinity dsDNA antibodies, a feature sometimes seen in lupus-like syndromes or non-specific systemic autoimmune activation. The extractable nuclear antigen (ENA) panel was negative, and rheumatoid factor was within normal limits, helping to exclude connective tissue diseases like rheumatoid arthritis or Sjögren’s syndrome (Table 1). Serum protein electrophoresis showed the absence of monoclonal bands and the presence of mildly elevated immunoglobulin levels, with a normal kappa/lambda ratio, pointing towards a polyclonal pattern of immune activation, lowering the suspicion for plasma cell dyscrasias such as multiple myeloma or monoclonal gammopathy of undetermined significance (Table 1). HIV, hepatitis B, and C serology were negative. Imaging with an abdominal ultrasound showed a normal-sized spleen, no lymphadenopathy, or secondary malignancy. A detailed medication history revealed no recent exposure to drugs frequently associated with warm AIHA. 

With a confirmed diagnosis of warm AIHA, treatment was initiated with high-dose oral prednisolone at 1 mg/kg/day with folic acid supplementation to support erythropoiesis. Proton pump inhibitors and oral calcium with cholecalciferol supplementation were given to reduce the risk of corticosteroid-induced gastric and osteoporotic complications. The patient later required two additional red blood cell transfusions, both administered under steroid cover, and extended antigen-matched blood products were used to minimize the risk of alloimmunization. His hemoglobin responded well to this approach, increasing to 98 g/L, after which he remained transfusion-independent and clinically stable at three months' follow-up. Ongoing monitoring was focused on hemolytic markers, including LDH, haptoglobin, reticulocyte count, and hemoglobin levels, to ensure sustained response and to detect any early signs of relapse. Given the absence of SLE features or hematological malignancy, and in the context of immune activation, the most likely etiology of warm AIHA in this case was immune dysregulation secondary to prostate cancer.

## Discussion

Warm AIHA is an immune-mediated condition characterized by the production of autoantibodies, typically IgG, that bind RBC surface antigens optimally at body temperature [7]. These antibody-coated erythrocytes are predominantly cleared through extravascular hemolysis in the spleen via Fc receptor-mediated phagocytosis by macrophages [7]. The risk factors of warm AIHA are heterogeneous, including idiopathic origins as well as associations with autoimmune diseases, infections, drug exposure, and malignancies [1,3,8]. In the context of solid tumors, the pathogenesis is thought to involve tumor-induced immune dysregulation [3]. Neoplastic cells can modify the immune microenvironment by promoting chronic antigenic stimulation, secreting pro-inflammatory cytokines, and impairing regulatory T-cell function. This dysregulation fosters polyclonal B-cell activation and a breakdown of peripheral self-tolerance, culminating in the production of pathogenic, high-affinity IgG autoantibodies targeting RBC antigens and resulting in immune-mediated hemolysis [3]. Warm AIHA is an uncommon but potentially serious condition that can be challenging to diagnose, particularly in older patients with multiple comorbidities.

Our case highlights several important diagnostic and clinical considerations. The patient presented with severe anemia without overt hemorrhage and normal iron studies, which initially supported a diagnosis of anemia of chronic disease. However, the subsequent transfusion was followed by biochemical markers of hemolysis and a strongly positive DAT, leading us to the diagnosis of AIHA.

Although the hemolysis followed red blood cell transfusion, the clinical and serological profile was inconsistent with a delayed hemolytic transfusion reaction (DHTR). There was no evidence of alloantibody formation, blood group incompatibility, or transfusion mismatch. The DAT was strongly positive for both IgG and complement (C3), supporting an autoimmune rather than alloimmune etiology [9]. Additionally, subsequent transfusions using extended antigen-matched blood were well tolerated under corticosteroid cover. The temporal association with transfusion may have unmasked an underlying autoimmune process rather than directly causing the hemolysis.

The patient’s autoimmune screen revealed positive ANA and markedly elevated anti-dsDNA by ELISA. However, the *Crithidia luciliae* assay was negative. This indirect immunofluorescence test detects high-affinity anti-dsDNA antibodies by binding to the kinetoplast of *Crithidia luciliae*, a protozoan containing pure circular DNA. A negative result suggests low-affinity, non-pathogenic antibodies, which can be present in individuals without clinical features of autoimmune disease, highlighting the potential for overdiagnosis when relying on serology alone [10]. In our patient, the presence of a positive ANA, elevated anti-dsDNA by ELISA, and polyclonal hypergammaglobulinemia raised the possibility of an evolving autoimmune condition such as SLE, although the absence of clinical features and a negative *Crithidia* assay argued against a definitive diagnosis [10,11]. There was no history of recent administration of medications known to trigger warm AIHA, including beta-lactam antibiotics or methyldopa [12].

Notably, no hematological malignancy was identified. While lymphoproliferative disorders are the most common malignancy associated with warm AIHA, there are occasional reports linking AIHA with solid tumors, including prostate cancer [4-6]. The pathogenesis in such cases may involve tumor-related immune dysregulation or paraneoplastic mechanisms [3]. Although rare, this possibility warrants consideration, especially when other causes have been ruled out. The case also highlights the importance of DAT testing in unexplained anemia and the need for careful transfusion planning in patients at risk of immune-mediated hemolysis [2]. Prompt immunosuppressive therapy with corticosteroids led to rapid clinical and hematological improvement in our patient. The patient’s clinical course highlights the importance of thorough diagnostic evaluation and tailored therapeutic intervention in warm AIHA, while also emphasizing the need to consider immune dysregulation in patients with solid organ malignancies presenting with hemolysis [1].

## Conclusions

This case highlights the diagnostic complexity of warm AIHA; while it is more commonly linked to lymphoproliferative malignancies or autoimmune diseases like lupus, this case shows that it can also occur as a rare immune complication of prostate cancer. The patient developed signs of hemolysis after transfusion, not because the transfusion caused it, but because it revealed an ongoing immune process. Although autoimmune tests showed positive ANA and dsDNA by ELISA, the negative *Crithidia* assay suggested these were low-affinity antibodies without strong clinical significance. This emphasizes the need to use specific confirmatory tests when interpreting autoantibodies. Early recognition through a positive DAT, prompt steroid treatment, and careful transfusion with antigen-matched blood led to a good outcome in this case. Clinicians should consider warm AIHA in any patient with unexplained anemia and hemolysis, even when systemic lupus or hematological malignancies are not present. This case highlights warm AIHA as a rare but important autoimmune complication of solid tumors, likely mediated by immune dysregulation. Greater recognition of this association may improve timely diagnosis and management.
